# Metabolites of soil microorganisms modulate amyloid β production in Alzheimer’s neurons

**DOI:** 10.1038/s41598-022-06513-z

**Published:** 2022-03-02

**Authors:** Takayuki Kondo, Tsuyoshi Yamamoto, Kaoru Okayama, Hideki Narumi, Haruhisa Inoue

**Affiliations:** 1 Center for iPS Cell Research and Application (CiRA), Kyoto University, Kyoto, 606-8507 Japan; 2 Medical-Risk Avoidance Based on iPS Cells Team, RIKEN Center for Advanced Intelligence Project (AIP), Kyoto, Japan; 3 iPSC-Based Drug Discovery and Development Team, RIKEN BioResource Research Center (BRC), Kyoto, Japan; 4 MicroBiopharm Japan Co., Ltd. JP, Nakagawara, Kiyosu, Aichi 452-0915 Japan; 5 Institute for Advancement of Clinical and Translational Science (iACT), Kyoto University Hospital, Kyoto, Japan

**Keywords:** High-throughput screening, Phenotypic screening, Alzheimer's disease, Soil microbiology, Induced pluripotent stem cells

## Abstract

Microbial flora is investigated to be related with neuropathological conditions in Alzheimer’s disease (AD), and is attracting attention as a drug discovery resource. However, the relevance between the soil microbiota and the pathological condition has not been fully clarified due to the difficulty in isolation culture and the component complexity. In this study, we established a library of secondly metabolites produced in microorganism to investigate the potential effect of microorganisms on the production of amyloid β (Aβ), one of the most representative pathogens of AD. We conducted a library screening to quantify Aβ and neuronal toxicity by using cortical neurons from human induced pluripotent stem cells (iPSCs) of AD patients after adding secondary metabolites. Screening results and following assessment of dose-dependency identified Verrucarin A, produced in *Myrothecium* spp., showed 80% decrease in Aβ production. Furthermore, addition of Mer-A2026A, produced in *Streptomyces pactum*, showed increase in Aβ42/40 ratio at the low concentration, and decrease in Aβ production at the higher concentration. As a result, established library and iPSC-based phenotyping assay clarified a direct link between Aβ production and soil microorganisms. These results suggest that Aβ-microorganism interaction may provide insight into the AD pathophysiology with potential therapeutics.

## Introduction

In 1928, Dr. Alexander Fleming discovered penicillin, the world’s first antibiotic, in the culture medium of *Penicillium notatum*^1^. After that, many antibiotics, hypercholesterolemia drugs, immunosuppressants, antitumor agents, etc., were isolated from microorganisms such as filamentous fungi and actinomycetes. At present, 60% of small molecules for therapeutic agents on the market are derived from natural products, and therefore secondary metabolites produced by microorganisms are important in the development of therapeutic drugs^2^. In the 1990s, combinatorial chemistry technology rapidly emerged, and produced a large array of structurally diverse compounds for high throughput screening^3^. As combinatorial chemistry technology has become more sophisticated, drug discovery based on natural products, including microorganisms, went downhill temporarily, and has received attention again in the 2000s^4,5^. As background for this revival, researchers re-acknowledged that many structures of the natural products are meant to be physiologically active^5^, and also hypothesized that there is nothing meaningless in nature. For example, bacteria or fungi, which are endosymbiont of plants (called “endophyte”), produce anti-fungal compounds to protect the host plant from exogenous harmful fungi, and are known to be an important resource for developing anti-fungal drugs^6^. Therefore, we had continued to collect and assemble a compound library of secondary metabolites, originating especially from soil microorganisms. When constructing the compound library from soil microorganisms, high-precision techniques and much labor are required to isolate microorganisms, to analyze compounds, and to maintain the library^7,8^. We have been developing drugs for decades using secondary metabolites produced by soil microorganisms, and maintain the database which covers information on the soil microorganism strains, optimized protocol for expansion culture, and nucleotide sequences of strains^9^. To maximize the potential of the library as drug candidates, we investigated the relationship between disease pathology and secondary metabolites of soil microorganisms, and tried to understand how microorganisms affect the pathology^10^.

In the 2000s, researchers focused on the connection between microbiota and diseases, and proved that microbiota of gut, oral cavity etc*.* are associated with the onset or progression of disease, such as brain disorders including Alzheimer’s disease (AD), one of the most common types of neurodegenerative disorders^11–14^. Regarding amyloid-β protein (Aβ), which is one of the most representative pathogens of AD, researchers have shown that microbiota accelerate the Aβ pathology through complex immune responses^11–14^. On the other hand, it has also been debated whether Aβ can be a protective molecule from the infection with microbiota including various viruses^15–19^. However, it remains unclear whether soil microorganisms can directly affect the production of Aβ in AD brain, or not. Therefore, we established a secondary metabolites library originating from soil microbiota, and applied the established library to the human-induced pluripotent stem cells (iPSCs) model which can recapitulate the Aβ metabolism, including altered Aβ production or Aβ42/40 ratio in iPSC-derived neurons from a patient with *PSEN1* mutation, and reasonable responsiveness to known Aβ-modifying agents^20,21^. Library screening clarified that one secondary metabolite accelerated and two metabolites could improve Aβ production in AD neurons. These results indicate that the established library of compounds derived from soil microorganisms will be a new human-cell-based approach for investigating therapeutic agents, and will provide a direct clue to understanding the interaction potential of Aβ in AD with microbiota.

## Results

### Establishment of compound library from soil-derived microorganisms

**Figure 1:**
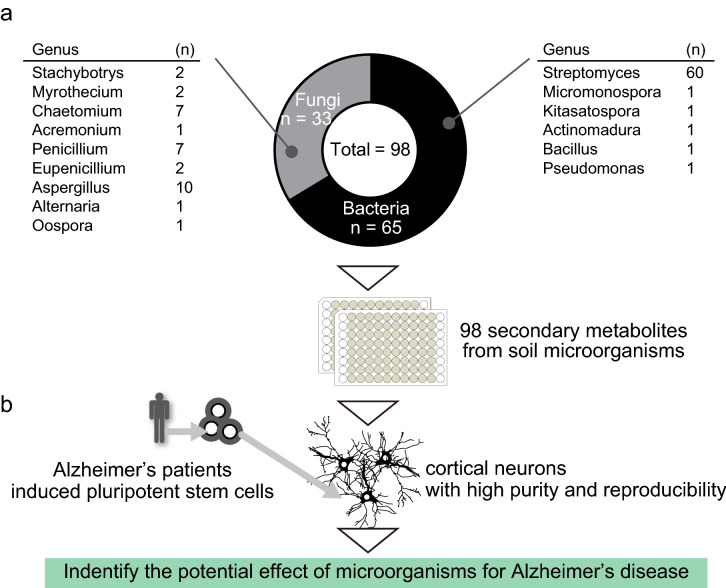
Screening system to evaluate compounds originating from soil microbiota. (**a**) Extraction and purification of compounds originating from soil microbiota. (**b**) Schema of study design and compound library from soil microbiota.

The relationship between microorganisms and diseases is clear in the research field of AD^11–14^. On the other hand, another research approach will be necessary to clarify how microorganisms are associated with diseases. Therefore, we have established compound libraries by regarding microorganisms as an attractive resource to investigate the kinds of compounds that modify diseases in order to understand how microorganisms alter pathological processes. Since the 1960s, we have endeavored to select microorganisms that produce industrially important fermentation products, such as compounds with powerful antibacterial and antitumor effects, from the microorganisms isolated from soil (Fig. 1a)^9^. In this study, we utilized a chemical library consisting of 98 compounds, all secondary metabolites originating from soil microbiota (Table 1, Fig. 1b).

**Table 1:** List of secondary metabolites from soil microorganisms.

No.	Compound	CAS no.	M.W.	Microorganisms by isolation culture
1	Desmycosin	11032-98-7	771.9	Streptomyces
2	Izenamicin B3	80240-61-5	581.7	Micromonospora
3	23-O-Demycinosyltylosin	79592-92-0	741.9	Streptomyces
4	Tylosin	1401-69-0	916.1	Streptomyces
5	5-O-Mycaminosyltylonolide	61257-02-1	597.7	Streptomyces
6	Demethylmacrocin	79404-98-1	888.0	Streptomyces
7	Macrocin	11049-15-3	902.1	Streptomyces
8	Carbomycin B	21238-30-2	826.0	Streptomyces
9	3-O-Acetyltylosin	63409-10-9	958.1	Streptomyces
10	Cytochalasin A	14110-64-6	477.6	Aspergillus
11	Cytochalasin B	14930-96-2	479.6	Aspergillus
12	Chaetoglobosin A	50335-03-0	528.6	Chaetomium globosum
13	Chaetoglobosin B	50335-04-1	528.6	Chaetomium globosum
14	Chaetoglobosin C	50645-76-6	528.6	Chaetomium globosum
15	Chaetoglobosin D	55945-73-8	528.6	Chaetomium globosum
16	Chaetoglobosin E	55945-74-9	530.7	Chaetomium globosum
17	Chaetoglobosin F	55945-75-0	530.7	Chaetomium globosum
18	Chaetoglobosin J	65745-47-3	512.6	Chaetomium globosum
19	Polyoxin D	22976-86-9	521.4	Streptomyces
20	Polyoxin L	22976-90-5	477.4	Streptomyces
21	Guanidylfungin A	94116-22-0	1130.5	Streptomyces
22	Mer-WF5027	147363-88-0	298.4	Aspergillus
23	Wortmannin	19545-26-7	428.4	Penicillium
24	Toyocamycin	606-58-6	291.3	Streptomyces
25	Amastatin	67655-94-1	474.6	Streptomyces
26	Griseolutein B	2072-68-6	344.3	Streptomyces
27	Relomycin	1404-48-4	918.1	Streptomyces
28	Calbistrin A	147384-55-2	540.7	Aspergillus
29	Neoviridogrisein II	66002-40-2	863.0	Aspergillus
30	Chrysomycin A	82196-88-1	508.5	Streptomyces
31	Gancidin W	5654-86-4	210.3	Streptomyces
32	Salinomycin	53003-10-4	751.0	Streptomyces
33	Leupeptin	55123-66-5	987.2	Streptomyces
34	Deferoxamine mesylate	138-14-7	656.8	Streptomyces
35	Pepstatin A	26305-03-3	685.9	Streptomyces
36	Chymostatin	9076-44-2	607.7	Streptomyces
37	Neoaspergillic acid	2152-59-2	224.3	Aspergillus
38	Brefeldin A	20350-15-6	280.4	Penicillium
39	Dehydrorabelomycin	30954-70-2	320.3	Streptomyces
40	Tenuazonic acid	610-88-8	197.2	Alternaria
41	Funiculosin	11055-06-4	491.6	Penicillium
42	Leucomycin U	31642-61-2	743.9	Streptomyces
43	3-O-Acetyl-4′′-O-isovaleryltylosin	63409-12-1	1042.3	Streptomyces
44	Leucomycin A1	16846-34-7	786.0	Streptomyces
45	Angolamycin	1402-83-1	916.1	Streptomyces
46	Mer-NF5003 E	159121-98-9	388.5	Stachybotrys
47	Stachybotrydial	149598-70-9	386.2	Stachybotrys
48	Mer-NF8054 A	157414-00-1	444.7	Aspergillus
49	Mer-A2026 B	144357-07-3	385.5	Streptomyces
50	Propioxatin A	102962-94-7	371.4	Kitasatospora
51	Cytomycin	2005-98-3	405.4	Pseudomonas
52	Bafilomycin A1	88899-55-2	622.8	Streptomyces
53	Leucanicidin	91021-66-8	783.0	Streptomyces
54	Bafilomycin B1	88899-56-3	816.0	Streptomyces
55	Bafilomycin D	98813-13-9	604.8	Streptomyces
56	Phenylacetic acid	103-82-2	136.2	Streptomyces
57	Eupenifeldin	151803-45-1	548.7	Eupenicillium
58	α-MAPI	70857-49-7	595.7	Streptomyces
59	Papulacandin E	61036-50-8	574.7	Streptomyces
60	Bacilycin	29393-20-2	270.3	Bacillus
61	Antibiotic SF 2487	120157-25-7	761.0	Actinomadura
62	β-MAPI	83830-01-7	611.7	Streptomyces
63	FK 506	104987-11-3	804.0	Streptomyces
64	Hikizimycin	12706-94-4	583.5	Streptomyces
65	Mithramycin	18378-89-7	1085.2	Streptomyces
66	Concanamycin A	80890-47-7	866.1	Streptomyces
67	Bredinin	50924-49-7	259.2	Eupenicillium
68	Fungichromin	6834-98-6	670.8	Streptomyces
69	Zincophorin	91920-88-6	568.8	Streptomyces
70	Verrucarin A	3148-09-2	502.6	Myothecium
71	Roridin A	14729-29-4	532.6	Myothecium
72	Mer-A2026 A	144357-08-4	413.6	Streptomyces
73	Efomycin A	106387-82-0	1039.3	Streptomyces
74	Boromycin	34524-20-4	879.9	Streptomyces
75	Pyridoxatin	135529-30-5	263.3	Acremonium
76	Complestatin	69598-75-0	1328.8	Streptomyces
77	Kalafungin	11048-15-0	300.3	Streptomyces
78	Blasticidin S	2079-00-7	422.4	Streptomyces
79	Gliotoxin G	53348-47-3	390.5	Aspergillus
80	Elaiophylin	37318-06-2	1025.3	Streptomyces
81	Efomycin G	114013-52-4	1011.3	Streptomyces
82	Medermycin	60227-09-0	457.5	Streptomyces
83	Oosporein	475-54-7	306.2	Oospora
84	Staurosporine	62996-74-1	466.5	Streptomyces
85	Citrinin	518-75-2	250.3	Penicillium
86	Spiculisporic acid	469-77-2	328.4	Penicillium
87	Calbistrin B	147384-56-3	540.7	Penicillium
88	5′-Deoxytoyocamycin	65562-55-2	275.3	Streptomyces
89	Aspergillin	490-02-8	224.3	Aspergillus
90	Chrysomycin B	83852-56-6	496.5	Streptomyces
91	Cephamycin C	34279-51-1	446.4	Streptomyces
92	Gliotoxin	67-99-2	326.4	Aspergillus
93	Streptothricin F	3808-42-2	502.5	Streptomyces
94	Penicillic acid	90-65-3	170.2	Penicillium
95	Streptothricin A	3484-67-1	1143.4	Streptomyces
96	Thiopeptin A1a	70591-36-5	1684.0	Streptomyces
97	Novobiocin	303-81-1	612.6	Streptomyces
98	Actinomycin D	50-76-0	1255.4	Streptomyces

### Screen for Aβ modifying compounds using the library of soil-derived microbiota

**Figure 2:**
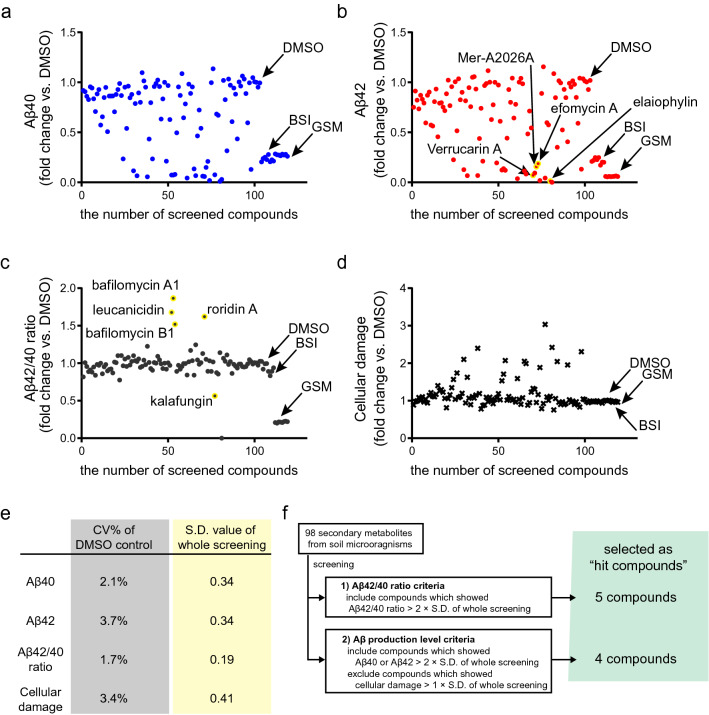
Primary screen results by using cortical neurons of sporadic Alzheimer’s disease. Scatter plot graph of (**a**) Aβ40 (blue round), (**b**) Aβ42 (red round), (**c**) Aβ42/40 ratio (black round), and (**d**) cellular damage (black x-mark). Fold change compared with DMSO control was plotted after adding compounds at a concentration of 1 μM. Hit compounds were highlighted by yellow color with compound name. *DMSO* dimethyl sulfoxide (negative control), *BSI* β-secretase inhibitor (β-secretase inhibitor IV, positive control for Aβ40), *GSM* γ-secretase modulator (JNJ-40418677, positive control for Aβ42 and Aβ42/40 ratio). (**e**) Table showing coefficients of variation (CV%) of DMSO, negative control (left column), and standard deviation (S.D.) of whole screening for each analyte. (**f**) Hit selection criteria.

To investigate the efficacy of soil-microbiota-derived compounds on AD pathology, we used iPSC-derived cortical neurons, which can recapitulate the Aβ phenotypes of AD. We utilized human iPSCs from patients with sporadic AD (SAD), and differentiated these iPSC into cortical neurons by temporary induction of human neurogenin 2 (NGN2) (Fig. 1b)^21^. We added 98 compounds originating from soil microbiota at a concentration of 1 μM, and quantified the concentrations of Aβ40 and Aβ42 in the culture medium to estimate the Aβ production from iPSC-derived neurons^20,21^. To evaluate the direct cellular damage by added compounds, we also quantified adenylate kinase spilled from dead and ruptured neurons in the culture medium^22,23^. The quantification of adenylate kinase in culture medium is widely used as cell-death indicator in compound screening, and has been applied to variable cell types as reproducible and high-sensitivity system^24,25^. We visualized the effect of compounds by plotting the alteration ratio to the dimethyl sulfoxide (DMSO) condition, set as negative controls (Fig. 2, Supplementary Table S1). To evaluate the assay stability within screening, we calculated the coefficient of variation (CV%) of DMSO controls (n = 5) for each outcome measure. Mean CVs for Aβ40, Aβ42, Aβ42/40 ratio, and cellular damage assay in DMSO condition were 2.1%, 3.7%, 1.7, and 3.4%, respectively (Fig. 2e, Supplementary Table S1). To minimize the effect of cellular damage on quantifying Aβ, we also conducted screening by using a 50 nM concentration of compounds, and found a similar tendency of alteration in Aβ production. We adopted the 1 μM concentration because the change in Aβ production was more evident (Fig. S2). From these results, we could confirm that this screening system successfully assessed dynamic Aβ responses and kept a low variability throughout the screening. As positive controls, we used known Aβ production-modifying compounds, including BACE inhibitor IV (β-secretase inhibitor: BSI) and JNJ-40418677 (γ-secretase modulator: GSM), and found that BSI and GSM reduced Aβ production (Fig. 2a,b) and suppressed the Aβ42/40 ratio (Fig. 2c). Selection criteria were set to identify hit compounds that alter Aβ production from the results of the screening assay (Fig. 2f). To leave out toxic compounds that could ostensibly lead to a decrease in Aβ production from fewer neurons, we excluded compounds causing more cellular damage than 1 × standard deviation (S.D.) of whole screening (Fig. 2e,f). To identify compounds that can modify the production of Aβ42, a toxic form of Aβ, we set cut-off thresholds for (1) increase or decrease in Aβ42 (> 2 × S.D. of whole screening), and for (2) increase or decrease in Aβ42/40 ratio (> 2 × S.D.). We identified nine hit compounds meeting the threshold criteria (Fig. 2f).

### Verrucarin A produced in *Myrothecium* spp. reduced Aβ production

**Figure 3:**
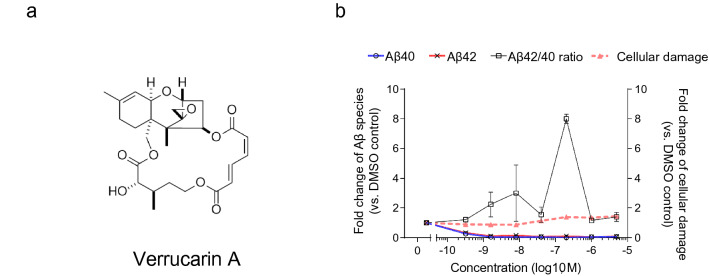
Verrucarin A reduced production without cellular damage. (**a**) Structure of Verrucarin A, (**b**) dose-dependent effect of Verrucarin A on Aβ40 (blue line), Aβ42 (red line), Aβ42/40 ratio (black line), and cellular damage (dash line). Data represent mean ± SD (n = 3 per clone).

To confirm the results of the first screening and estimate the effective concentration of hit compounds, we investigated whether dose-dependent reactivity to these compounds could be seen by preparing a dilution series, including 0, 0.00032, 0.0016, 0.0080, 0.040, 0.20, 1.0, 5.0 μM of each compound. We quantified the alteration ratio of Aβ species or cellular damage after 48 h of treatment with nine hit compounds in the first screening. All compounds clearly showed the Aβ modifying effect, observed with 1 μM concentration like in the first screening, and also showed a dose-dependent effect on Aβ production or cellular damage. Treatment with Verrucarin A (Fig. 3a) reduced Aβ40 and Aβ42 production even at the low concentration (0.32, 1.6, 8 nM) without cellular damage (Fig. 3b). Higher concentration of Verrucarin A (0.2–5 μM) showed weak cellular damage, whose relative value was a 1.35- to 1.45-fold increase compared to DMSO negative control. Verrucarin A was isolated from soil *Myrothecium* spp., and was known as a trichothecene antibiotic, like macrolide antibiotic, or anticancer therapeutics by inhibiting protein biosynthesis by preventing peptidyl transferase activity^26^. Verrucarin A was also reported to be toxic in some cell types or to cause contact dermatitis^27^. However, the system based on iPSC-derived neurons in this study did not show any harmful events with treatment by Verrucarin A. These results indicate that Verrucarin A produced in *Myrothecium* spp. could be a potential therapeutic agent for reducing Aβ after optimizing the chemical structure for drug development.

### Mer-A2026A produced in *Streptomyces* spp. showed two-sided effect on Aβ production

**Figure 4:**
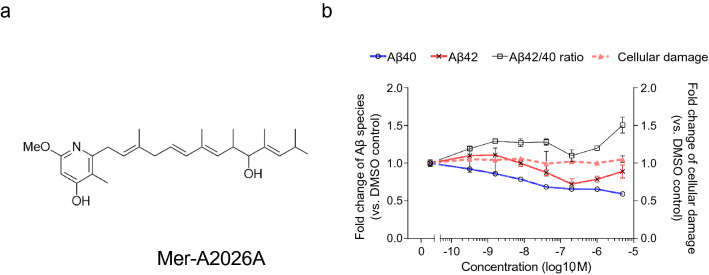
Mer-A2026A upregulated Aβ42/40 ratio at low concentration and reduced Aβ production at high concentration. (**a**) Structure of Mer-A2026A, (**b**) dose-dependent effect of Mer-A2026A on Aβ40 (blue line), Aβ42 (red line), Aβ42/40 ratio (black line), and cellular damage (dash line). Data represent mean ± SD (n = 3 per clone).

Treatment with Mer-A2026A (Fig. 4a) showed increased Aβ42/40 ratio and decreased Aβ40, protective Aβ species even at low concentrations from 1.6 to 200 nM (Fig. 4b). Mer-A2026A was isolated from soil *Streptomyces* spp., including *Streptomyces kamatakensis* or *Streptomyces pactum*, and is known as the vasodilator^28^. This result indicated that *Streptomyces kamatakensis* or *Streptomyces pactum* producing Mer-A2026A can be a risk factor for the amyloid burden observed in the AD brain. On the other hand, high concentration of Mer-A2026A (0.04–5 μM) reduced both Aβ40 and Aβ42 with limited cellular damage down to the half level of DMSO control. However, treatment with 1–5 μM Mer-A2026A showed increased trend in Aβ42 production level, comparing with 0.2 μM like biphasic dose–response. It was reported that a previous genomic cohort with a half reduction in Aβ40 or Aβ42 could be a protective factor for the onset or progression of AD^29^. Therefore, efficient delivery of Mer-A2026A into the brain could be future preventive medicine. There results showed that *Streptomyces kamatakensis* or *Streptomyces pactum* has the dual nature of risk and prevention in AD.

For the six compounds, including leucanicidin, bafilomycin A1, roridin A, bafilomycin B1, kalafungin, and efomycin A, other than Verrucarin A, a Mer-A2026A, cellular damage was observed even in the low concentration range of 0.32–8 nM, so it was difficult to judge the change in Aβ production level because smaller numbers of neurons in a culture well can produce only a small dose of Aβ (Fig. S3). In particular, elaiophylin also showed cellular damage and reduction in Aβ production at the same concentration, from 200 to 5 μM.

### Different responsiveness to familial AD neurons

We conducted screening and dose-dependency analysis by using the cortical neurons of a SAD patient to investigate the environmental effect on the general AD pathology. Occasionally, it has been reported that neurons of SAD and familial AD (FAD) have different reactivity to compounds. To investigate this issue, we used previously established iPSCs of a patient with FAD bearing *PSEN1* G384A mutation^21^. We analyzed the dose-dependency of 9 hit compounds, identified in screening by the use of FAD neurons. The dose-dependency curves of FAD neurons were similar to those of SAD neurons (Fig. S4). In particular, 0.00032 or 0.0016 μM Mer-A2026A reduced the Aβ40 and Aβ42 production in contrast with the result when using SAD neurons. These results indicate that identified secondary metabolites can affect not only the Aβ production of SAD neurons, but also that of FAD neurons. Furthermore, to investigate whether secondary metabolites have different effects on Aβ production for each individual or for different pathological conditions, we evaluated the dose-dependency of Verrucarin A and Mer-A2026A by using four additional iPSCs established from two healthy controls, another SAD patient, and another familial AD patient with APP V717L mutation (APP-FAD)^21^ (Fig. S5). We investigated the dose-dependent curve of the alteration in Aβ production or cellular damage when adding Verrucarin A by using healthy controls, SAD, and APP-FAD iPSCs-derived neurons. Although each clone has certain characteristics, Verrucarin A also tended to suppress Aβ production regardless of the pathological conditions of healthy control, SAD, and FAD. Like the use of other SAD and FAD clones, Mer-A2026A also tends to increase Aβ42/40 ratio in the low concentration range and suppress Aβ production in the high concentration range for neurons with different pathological conditions. Taken together, these results suggest that Verrucarin A and Mer-A2026A alter Aβ production regardless of individual or pathological differences. From these results, we could identify Verrucarin A, produced in Myrothecium spp., and Mer-A2026A, produced in Streptomyces kamatakensis or Streptomyces pactum, as a modifier of Aβ production in AD neurons.

## Discussion

In this study, we constructed a screening assay by using cortical neurons from AD patient iPSCs, and evaluated the effect of secondary metabolites of soil microorganisms on Aβ production. Following investigation for dose-dependent responses of hit compounds, we identified second metabolites and metabolite-related microorganisms as therapeutic or risk agents for AD pathophysiology.

Microorganisms have become known to be associated with the onset and progression of AD. In particular, microorganisms in the oral cavity and intestinal tract are known to be epidemiologically associated with various AD pathological conditions, and it is expected that understanding and control of microorganisms can be a therapeutic approach^12^. As a background to this, the exposure situation to soil microorganisms changes according to lifestyle, even within the same individual, depending on age, place of residence, and the like. Therefore, it is difficult to epidemiologically clarify the relationship between a specific disease and soil microorganisms. On the other hand, we clarified the effects of soil microorganisms on pathological conditions through the secondary metabolites produced by soil microorganisms, and the presented approach in this study can be one of the solutions for understanding the impact of microorganisms on the disease pathophysiology. Until now, it has not been clear whether exactly the same compounds as the secondary metabolites identified in this study will be detected in the human body. In addition, secondary metabolites are probably not unique to each microbial species. However, secondary metabolites with similar structures or functions may be produced in the human body by microorganisms of the same genus and affect Aβ dynamics. Connecting these points, investigation for the microbiome in the human body provide the key information. In this regard, accumulated microbiome datasets also proved the incursion of various bacteria including actinobacteria into central nervous systems by using 16S ribosomal gene sequencing of AD brain extracts^30^. In this study, we used SAD iPSCs for the screening. In the future, by using a larger cohort of iPSCs, it will be possible to investigate the versatility of responsiveness to the compounds identified in this study. It also may be possible to clarify the kinds of populations that are susceptible to the effect of soil microorganisms and thereby lead to the development of SAD. The combination of our in vitro system and microbiome datasets will help us to understand the role of microbiota on AD.

Aβ is widely understood to be a harmful substance for neural function. On the other hand, researchers also hypothesized that Aβ actually plays a bio-defensive role via the immune system, and that Aβ is deposited as a result of infection and inflammatory events^15–19^. From this point of view, secondary metabolites of soil microorganisms, which reduce Aβ, can be a weapon to break through the Aβ-based defense system. In order for Aβ to act as a defense against microorganisms, it is important to form fibrils that can catch microbes via eventual entrapment of unattached microbes^15^. Therefore, investigating whether secondary metabolites affect not only Aβ production but also fibril formation is of particular interest. In the future, a system to quantify the extracellular depositions of Aβ fibrils will provide information on these insights. These current data in this study may unravel a part of the evolutionary history of humankind when handling Aβ as a trade-off between defense-system and neuro-toxic agents.

The epidemiological approach of microorganisms can analyze a large number of patient samples by metagenomic approach and investigate the relevance between the pathological condition and microbiome as summation of microbial abundance^31,32^. Therefore, it is not difficult to know which specific microorganism or substances affects the pathological condition directly. In this study, we converted microorganisms to secondary metabolites to link them with the functional pathology based on human cells, and this approach enables us to search for the kind of microorganisms that impact certain kinds of pathological conditions directly. In addition, we cannot adapt the results of this study to the brain environment of human beings because we have no direct evidence to confirm that nano-molar to micro-molar concentrations of secondary metabolites exist at a natural level in the human brain. Therefore, the combination of iPSC-based model and animal models will open a way to understanding the relationship between AD and microorganisms.

## Conclusion

In future study, we plan to carry out research using various pathological phenotypes and soil microorganisms to deeply explore the relationship among them. At the same time, we would like to connect the presented system to research on pathological relevance as a more complex soil microbiome, including a metagenomic database of soil microorganisms, which is being carried out with international cooperation. Finally, we hope that the microorganisms in the soil will lead to an understanding of the pathology, and provide preventive or therapeutic solutions.

## Experimental procedures

### Ethical approval

All experimental protocols in the study were approved by the ethics committee of the Graduate School and Faculty of Medicine Kyoto University (R0091 and G259). The study was performed conforming to the guidelines of the Declaration of Helsinki and conducted after written informed consent was obtained from all participants.

### Preparation of secondary metabolites extracted from soil microorganisms

The producing microorganisms were isolated from various kinds of environmental resource, e.g., soil, plants, etc., and were cultivated in 250-mL Erlenmeyer flasks, each containing 25 mL of a seed medium as previously described, depending on the different types of species^33–38^. The whole-culture broth was extracted with an equal volume of n-BuOH, and was fractionated by using silica gel medium-pressure liquid chromatography (MPLC). After step-wise filtration steps, crude materials were purified by HPLC and stocked as extracted compounds.

### Establishment of human induced pluripotent stem cells

For the establishment of human induced pluripotent stem cells from peripheral blood mononuclear cells (PBMCs), human cDNAs for reprogramming factors were transduced in human PBMCs with episomal vectors (SOX2, KLF4, OCT4, L-MYC, LIN28, dominant negative p53). Several days after transduction, PBMCs were harvested and replated on iMatrix-coated dishes. On the following day, the medium was changed to StemFit AK03. After that, the medium was changed every other day by using StemFit AK02N. Twenty days after transduction, iPSC colonies were picked up. Established PBMC-origin iPSCs were expanded for neural differentiation^21^.

### Generation of iN-iPSCs

To establish a robust, quick differentiation method, we utilized direct conversion technology. Human neurogenin2 (NGN2) cDNA, under tetracycline-inducible promoter (tetO), was transfected into iPSCs by a *piggyBac* transposon system and Lipofectamine LTX (Thermo Fisher Scientific Inc., Waltham, MA). We used the vector containing tetO::NGN2 After antibiotic selection of G418 disulfate (Nacalai-Tesque, Kyoto, Japan), we picked out colonies and selected subclones that could efficiently differentiate into neurons by inducing the temporal expression of NGN2, with MAP2/DAPI 96% < purity^21^ as iN-iPSCs.

### Screening assay of secondary metabolites from soil microorganisms

On day 0, iN-iPSCs were dissociated with TrypLE express (Gibco, Thermo Fisher Scientific Inc.) and disseminated on a mixed coating of poly-l-lysine (final 0.0002% v/w, Sigma Aldrich, Japan), Symthemax II-SC (final 20 μg/mL, Corning, NY), and Matrigel (final 2% v/v, Corning). Disseminated iPSCs were cultured in Neurobasal Medium (Gibco, Thermo Fisher Scientific Inc.), supplemented with 0.5% B27 without Vitamin A (Gibco, Thermo Fisher Scientific Inc.), 1 × Glutamax (Gibco, Thermo Fisher Scientific Inc.), 2 μg/mL doxycycline hydrochloride (Wako Pure Chemicals Industries, Ltd., Japan), and 10 μM Y-27632 (Nacalai-Tesque) from day 0 to day 5. On day 5, differentiated neural cells were disseminated into 96-well plates with the function as passive humidity control (Edge plate, Nunc, Thermo Fisher Scientific Inc.), which can eliminate evaporation of culture medium and minimize well-to-well variability. Disseminated neural cells were cultured in Neurobasal Medium (Gibco, Thermo Fisher Scientific Inc.), supplemented with 0.5% B27 without Vitamin A (Gibco, Thermo Fisher Scientific Inc.) and 1 × Glutamax (Gibco, Japan) from day 5 to day 8. On day 8, all culture medium was replaced with 120 μL fresh medium, containing each of the 1 μM compounds in final 0.1% DMSO carrier. For negative control, all culture medium was replaced with 120 μL fresh medium, containing only 0.1% DMSO carrier. Neurons or culture media were subjected to analysis 48 h later. Each 96-well plate contained 80 or 18 secondary metabolites per plate, four positive controls for Aβ40 (2 μM β-secretase inhibitor IV), four positive controls for Aβ42 and Aβ42/40 ratio (2 μM JNJ-40418677), and three or two negative controls (0.1% DMSO carrier). The raw data of each compound or positive control was normalized to calculate the alteration ratio by using the average data of five DMSO controls [fold change = raw data of each compound/averaged data of five DMSO controls] (Supplementary Table S1).

### Electrochemiluminescence assays for Aβ

Aβ species in culture media were measured by human (6E10) Aβ 3-Plex Kit (Meso Scale Discovery, Rockville, MD) for extracellular human Aβ. For Aβ species, this assay uses 6E10 antibody to capture Aβ peptide and SULFO-TAG-labeled different C-terminus specific anti-Aβ antibodies for detection by electrochemiluminescence with Sector Imager 2400 (Meso Scale Discovery). Quantified Aβ values were adjusted using total protein concentration of neurons and compared among conditions.

### Assay for cellular damage

We evaluated the cellular damage 48 h after the addition of compounds for screening assay. For quantification of cellular damage, we collected the supernatant and measured the signal counts of adenylate kinase (ToxiLight bioassay kit, Lonza, Basel, Switzerland), originated from damaged cells.

### Evaluation of dose-dependency

To evaluate the dose-dependency of secondary metabolites on Aβ production, we made 1/5 dilution series for each compound. First, we made a 5-μM solution of each compound in 0.1% DMSO-containing culture medium. Next, we sequentially diluted the 5-μM solution to 5 times its volume by using 0.1% DMSO-containing culture medium, and prepared 0.00032, 0.0016, 0.0080, 0.040, 0.20, 1.0, 5.0 μM of compound. We also set the 0.1% DMSO-containing culture medium as negative control.

### Statistical analysis

All data and graphs were plotted by Prism 7 (GraphPad Software Inc., La Jolla, CA). The calculation of CV% or standard deviation was performed by using Microsoft Excel 2016 (Microsoft, Redmond, WA).

## Supplementary Information

Supplementary Information.
