# Supplementary material for: Metabolites of soil microorganisms modulate amyloid β production in Alzheimer’s neurons
Source: Sci Rep. 2022 Mar 2;12:2690. doi: 10.1038/s41598-022-06513-z (PMC8891331; doi:10.1038/s41598-022-06513-z)
Supplement: Supplementary file 1 — Supplementary Information. [file 41598_2022_6513_MOESM1_ESM.pdf]

Figure S1

Kondo et al.

a

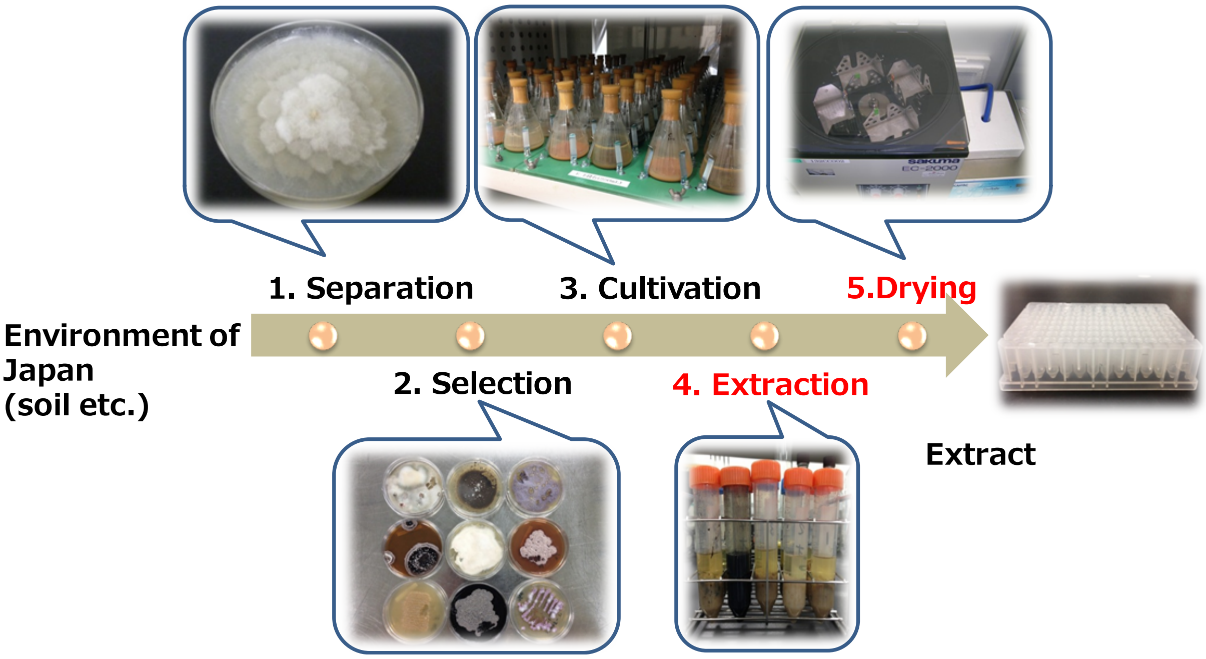

Figure S1

Schema of isolation culture of microbiota

Figure S2

Kondo et al.

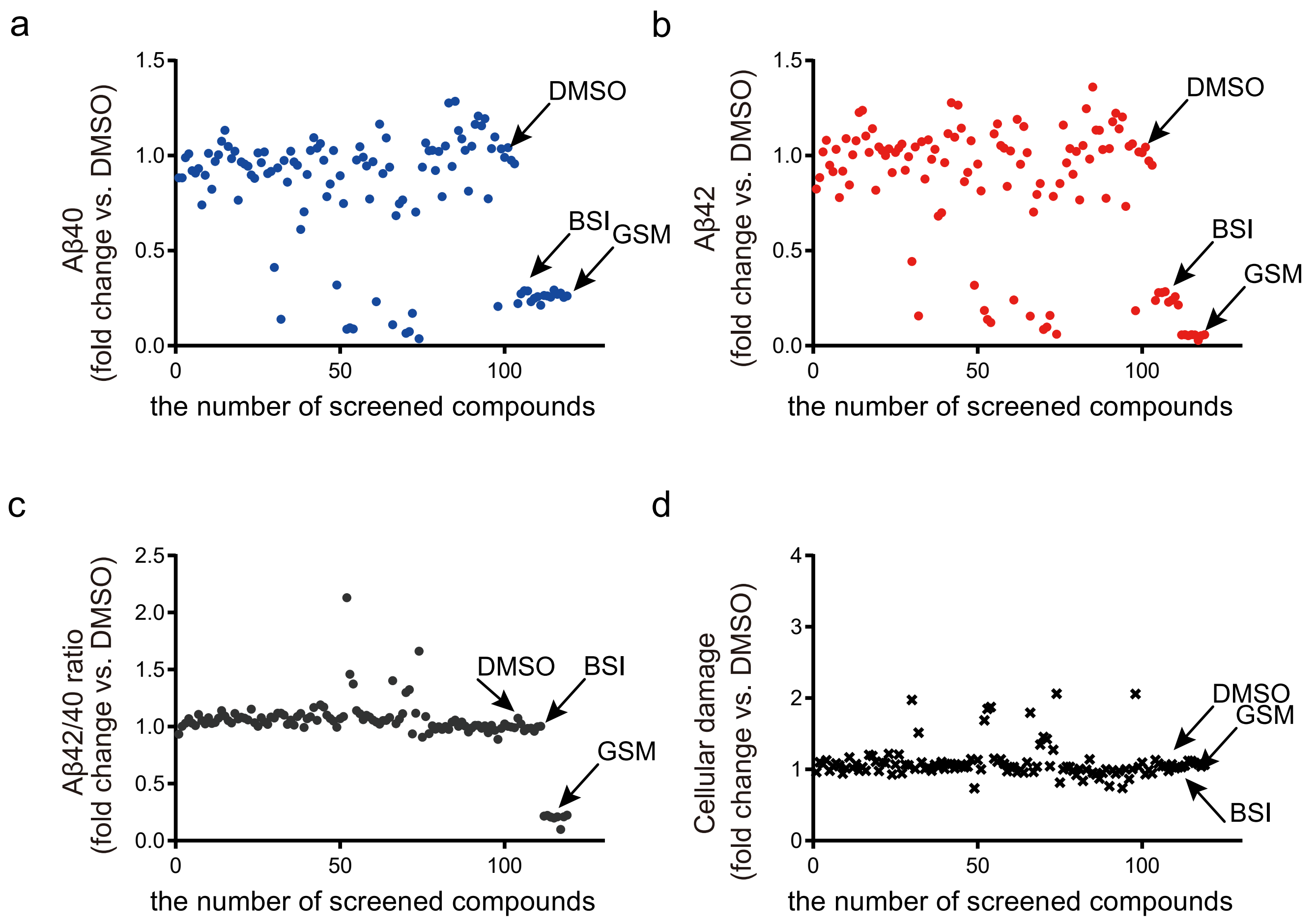

Figure S2

Primary screen results by using cortical neurons of familial Alzheimer's disease

Scatter plot graph of (a) Aβ40 (blue round), (b) Aβ42 (red round), (c) Aβ42/40 ratio (black round), and (d) cellular damage (black x-mark). Fold change compared with DMSO control was plotted after adding compounds at a concentration of 50 nM. DMSO: dimethyl sulfoxide (negative control); BSI: β-secretase inhibitor (β-secretase inhibitor IV, positive control for Aβ40); GSM: γ-secretase modulator (JNJ-40418677, positive control for Aβ42 and Aβ42/40 ratio).

Figure S3

Kondo et al.

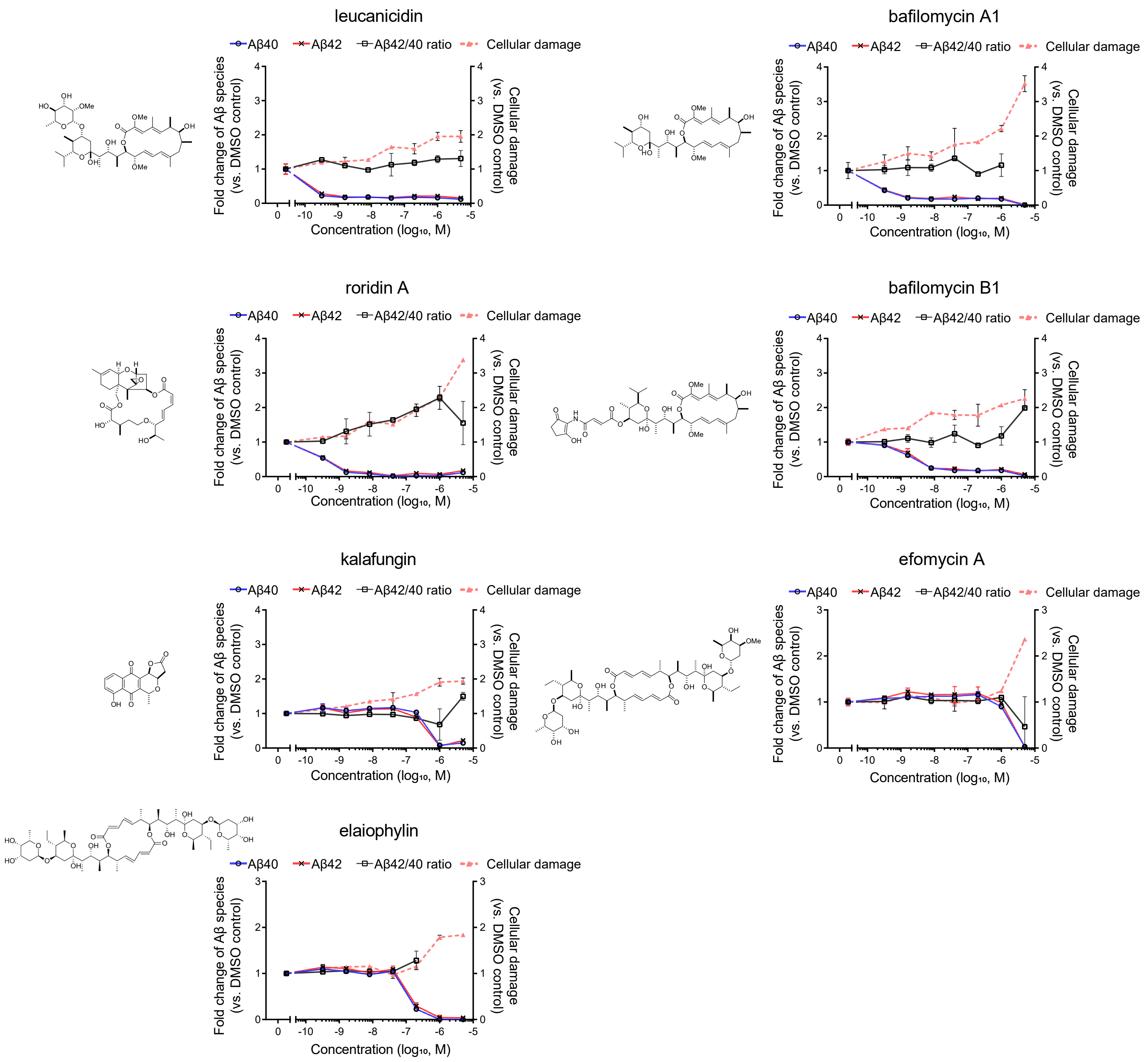

Figure S3

Evaluation of dose-dependent effect of hit compounds from the screening assay by using iPSCs of a patient with sporadic AD.

The effect on Aβ40 (blue line), Aβ42 (red line), Aβ42/40 ratio (black line), and cellular damage (dash line) was shown for each compound. Data represent mean ± SD (n = 3 per clone).

Figure S4

Kondo et al.

Verrucarin A

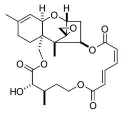

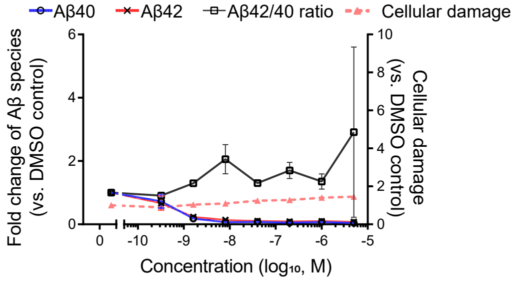

Mer-A2026A

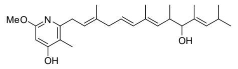

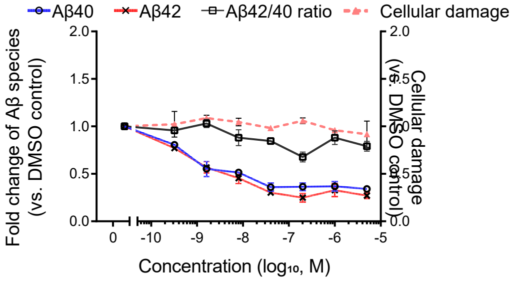

leucanicidin

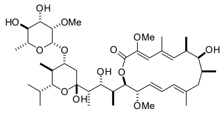

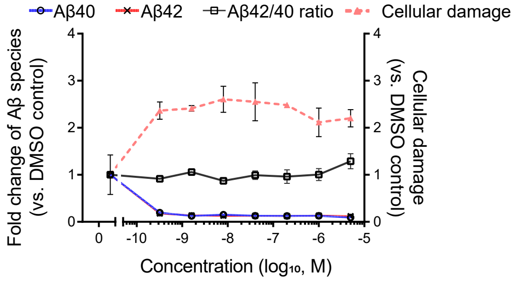

bafilomycin A1

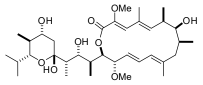

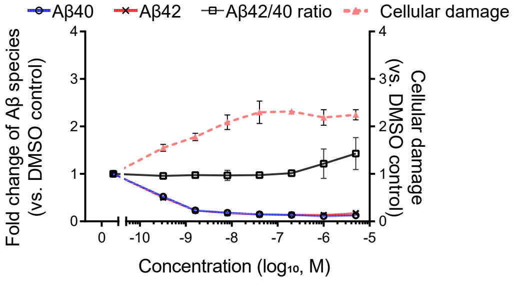

roridin A

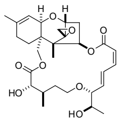

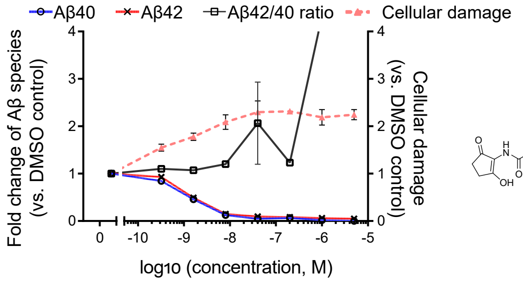

bafilomycin B1

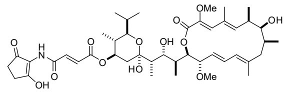

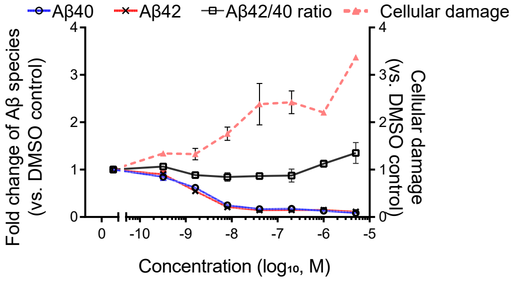

kalafungin

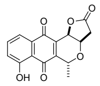

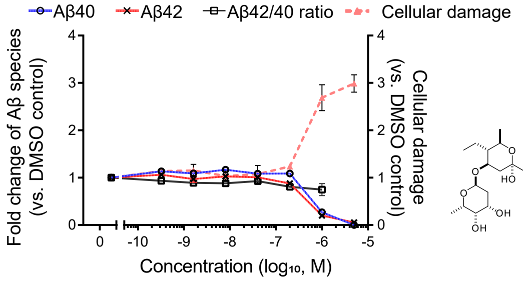

efomycin A

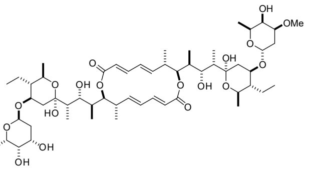

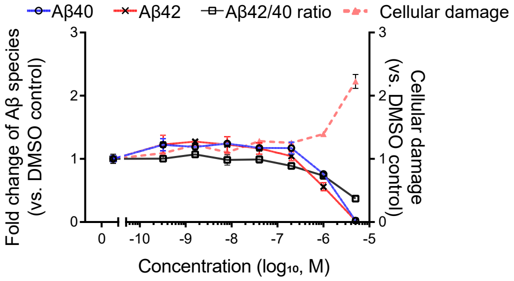

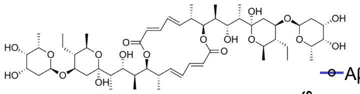

elaiophylin

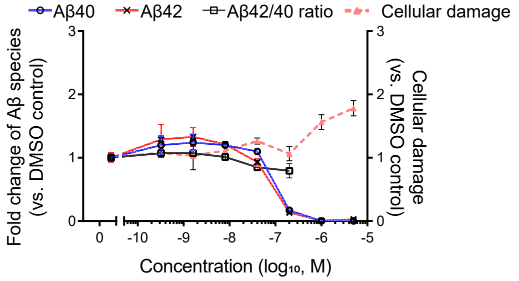

Figure S4

Evaluation of dose-dependent effect of hit compounds from the screening assay by using iPSCs of a patient with familial AD bearing *PSEN1* G384A mutation.

The effect on Aβ40 (blue line), Aβ42 (red line), Aβ42/40 ratio (black line), and cellular damage (dash line) was shown for each compound. Data represent mean ± SD (n = 3 per clone).

Figure S5

Kondo et al.

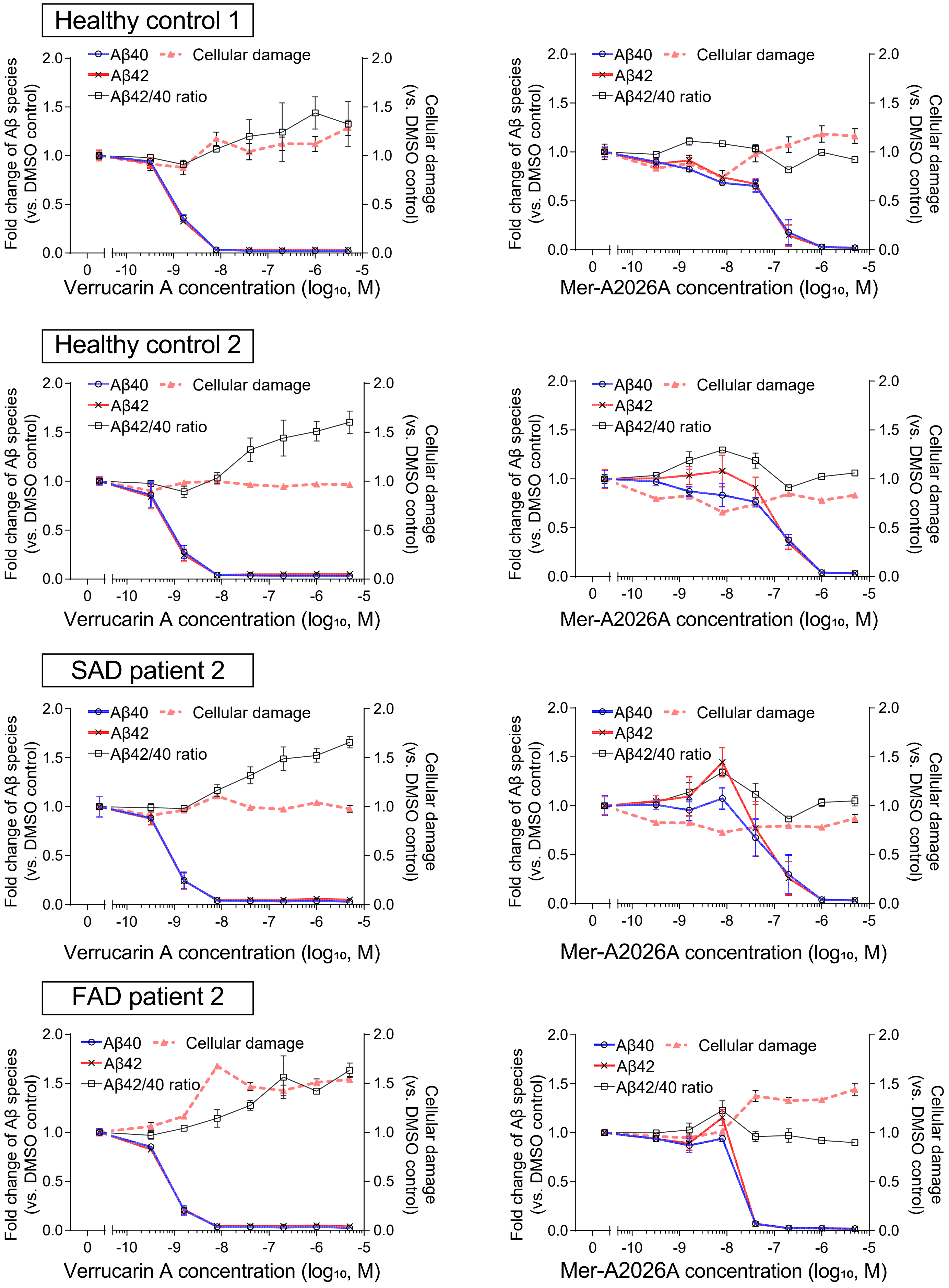

Figure S5

Evaluation of dose-dependent effect of Verrucarin A and Mer-A2026A by using iPSCs of healthy controls (Healthy control 1 and Healthy control 2), another patient of sporadic AD (SAD patient 2), and another patient of familial AD bearing *APP* V717L mutation (FAD patient 2)

The effect on Aβ40 (blue line), Aβ42 (red line), Aβ42/40 ratio (black line), and cellular damage (dash line) was shown for each compound. Data represent mean ± SD (n = 3 per clone).

Supplementary table S1: Results of the first screening based on neurons with SAD

No.	Compound	Aβ40	Aβ42	Aβ42/40 ratio	toxicity	Hit criteria
1	desmycosin	0.919	0.751	0.818	0.886	-
2	izenamicin B3	0.907	0.913	1.007	0.980	-
3	23-O-demycinosyltylosin	0.860	0.822	0.956	0.995	-
4	tylosin	0.829	0.735	0.887	1.084	-
5	5-O-mycaminosyltylonolide	0.956	0.870	0.910	1.003	-
6	demethylmacrocin	0.943	0.831	0.881	1.105	-
7	macrocin	0.835	0.793	0.949	0.970	-
8	carbomycin B	0.708	0.594	0.839	1.041	-
9	3-O-acetyltylosin	0.885	0.871	0.984	0.941	-
10	cytochalasin A	0.929	0.903	0.972	1.137	-
11	cytochalasin B	0.573	0.573	1.000	1.216	-
12	chaetoglobosin A	0.655	0.563	0.859	1.120	-
13	chaetoglobosin B	0.848	0.807	0.952	1.107	-
14	chaetoglobosin C	0.949	0.933	0.983	1.170	-
15	chaetoglobosin D	0.507	0.455	0.897	1.401	-
16	chaetoglobosin E	0.863	0.792	0.917	1.265	-
17	chaetoglobosin F	0.863	0.797	0.924	1.296	-
18	chaetoglobosin J	0.864	0.753	0.871	1.228	-
19	polyoxin D	0.706	0.717	1.015	1.060	-
20	polyoxin L	0.879	0.885	1.007	0.812	-
21	guanidylfungin A	0.928	1.022	1.101	1.055	-
22	Mer-WF5027	0.988	0.766	0.776	1.158	-
23	wortmannin	0.868	0.957	1.102	1.349	-
24	toyocamycin	**0.234**	**0.226**	0.965	**1.570**	-
25	amastatin	0.952	1.006	1.057	1.040	-
26	griseolutein B	0.539	0.516	0.957	**1.738**	-
27	relomycin	0.961	1.036	1.077	1.082	-
28	calbistrin A	0.845	0.825	0.977	1.112	-
29	neoviridogrisein II	0.879	0.901	1.025	1.198	-
30	chrysomycin A	**0.115**	**0.127**	1.100	**2.103**	-
31	gancidin W	0.788	0.815	1.035	0.942	-
32	salinomycin	**0.066**	**0.067**	1.022	**1.626**	-
33	leupeptin	0.894	0.946	1.058	1.068	-
34	deferoxamine mesylate	0.801	0.756	0.944	0.896	-
35	pepstatin A	1.097	1.051	0.958	1.113	-
36	chymostatin	1.015	1.057	1.041	1.071	-
37	neoaspergillic acid	0.952	0.933	0.980	1.152	-
38	brefeldin A	**0.056**	**0.069**	1.225	**2.398**	-
39	dehydrorabelomycin	0.433	0.438	1.011	1.190	-
40	tenuazonic acid	0.797	0.741	0.930	0.783	-
41	funiculosin	**0.234**	**0.212**	0.905	**1.871**	-
42	leucomycin U	0.959	0.968	1.009	0.887	-
43	3-O-acetyl-4"-O-isovaleryltylosin	0.988	0.988	1.001	1.091	-
44	leucomycin A1	1.024	1.117	1.092	0.973	-
45	angolamycin	0.958	1.002	1.046	1.095	-
46	Mer-NF5003 E	0.633	0.628	0.993	1.009	-
47	stachybotrydial	0.835	0.776	0.930	0.921	-
48	Mer-NF8054 A	1.000	1.000	1.000	1.036	-
49	Mer-A2026 B	**0.211**	**0.195**	0.920	**1.579**	-
50	propioxatin A	0.969	0.974	1.005	1.189	-
51	cytomycin	0.629	0.610	0.970	1.023	-
52	bafilomycin A1	**0.074**	**0.123**	**1.677**	**1.531**	1
53	leucanicidin	**0.074**	**0.137**	**1.866**	**1.709**	1
54	bafilomycin B1	**0.079**	**0.120**	**1.518**	**2.071**	1
55	bafilomycin D	0.496	0.442	0.891	1.014	-
56	phenylacetic acid	0.955	0.929	0.973	1.111	-
57	eupenifeldin	0.869	0.800	0.920	1.052	-
58	α-MAPI	1.087	1.042	0.958	1.010	-
59	papulacandin E	0.646	0.643	0.996	1.038	-
60	bacilycin	0.785	0.781	0.995	0.964	-
61	antibiotic SF 2487	**0.043**	**0.036**	0.843	**1.592**	-
62	β-MAPI	0.982	1.001	1.019	1.039	-
63	FK 506	0.889	1.040	1.170	0.904	-
64	hikizimycin	0.954	0.986	1.034	1.080	-
65	mithramycin	**0.134**	**0.113**	0.842	**2.053**	-
66	concanamycin A	**0.067**	**0.083**	1.246	**1.991**	-
67	bredinin	0.464	0.484	1.044	0.881	-
68	fungichromin	0.723	0.713	0.986	0.950	-

69	zincophorin	0.540	0.544	1.008	1.230	**-**
70	verrucarin A	**0.062**	**0.070**	1.129	1.329	**2**
71	roridin A	**0.058**	**0.094**	**1.620**	1.301	**1**
72	Mer-A2026 A	**0.150**	**0.157**	1.052	0.953	**2**
73	efomycin A	**0.209**	**0.187**	0.893	1.165	**2**
74	boromycin	**0.018**	**0.018**	1.033	**1.884**	**-**
75	pyridoxatin	0.997	0.889	0.892	0.788	**-**
76	complestatin	1.136	1.156	1.018	0.926	**-**
77	kalafungin	**0.076**	**0.043**	**0.564**	**3.036**	**1**
78	blasticidin S	0.683	0.597	0.874	1.100	**-**
79	gliotoxin G	0.671	0.574	0.856	1.010	**-**
80	elaiophylin	**0.010**	**0.011**	1.141	1.119	**2**
81	efomycin G	**0.029**	**0.000**	**0.000**	**2.423**	**-**
82	medermycin	1.019	1.087	1.067	0.756	**-**
83	oosporein	0.827	0.847	1.025	1.057	**-**
84	staurosporine	0.316	0.351	1.112	**2.045**	**-**
85	citrinin	1.033	1.016	0.983	0.960	**-**
86	spiculisporic acid	0.972	0.994	1.023	0.902	**-**
87	calbistrin B	0.607	0.525	0.864	0.970	**-**
88	5'-deoxytoyocamycin	0.943	0.930	0.986	0.896	**-**
89	aspergillin	0.882	0.847	0.960	0.928	**-**
90	chrysomycin B	0.434	0.406	0.935	**1.955**	**-**
91	cephamycin C	1.004	0.990	0.987	1.025	**-**
92	gliotoxin	0.964	0.953	0.989	0.961	**-**
93	streptothricin F	1.063	1.015	0.955	0.991	**-**
94	penicillic acid	1.034	0.981	0.949	0.901	**-**
95	streptothricin A	0.889	0.850	0.956	0.971	**-**
96	thiopeptin A1a	0.595	0.590	0.992	0.887	**-**
97	novobiocin	0.995	1.042	1.047	0.833	**-**
98	actinomycin D	**0.129**	**0.132**	1.023	**2.310**	**-**
NA	DMSO (negative control)	1.007	1.035	0.988	1.045	**-**
NA	DMSO (negative control)	1.006	1.013	1.007	1.029	**-**
NA	DMSO (negative control)	1.001	1.004	1.003	0.982	**-**
NA	DMSO (negative control)	0.952	0.928	0.975	0.948	**-**
NA	DMSO (negative control)	0.994	1.020	1.026	0.997	**-**
NA	BACE inhibitor IV (BSI)	**0.205**	**0.210**	1.023	0.979	**-**
NA	BACE inhibitor IV (BSI)	**0.238**	**0.219**	0.921	0.969	**-**
NA	BACE inhibitor IV (BSI)	**0.250**	**0.248**	0.992	0.985	**-**
NA	BACE inhibitor IV (BSI)	**0.228**	**0.227**	0.996	0.995	**-**
NA	BACE inhibitor IV (BSI)	**0.279**	**0.248**	0.891	0.963	**-**
NA	BACE inhibitor IV (BSI)	**0.206**	**0.171**	0.833	0.982	**-**
NA	BACE inhibitor IV (BSI)	**0.226**	**0.201**	0.893	0.936	**-**
NA	BACE inhibitor IV (BSI)	**0.214**	**0.201**	0.939	0.990	**-**
NA	JNJ-40418677 (GSM)	**0.285**	**0.059**	**0.209**	1.007	**-**
NA	JNJ-40418677 (GSM)	**0.276**	**0.056**	**0.201**	1.000	**-**
NA	JNJ-40418677 (GSM)	**0.273**	**0.059**	**0.218**	1.012	**-**
NA	JNJ-40418677 (GSM)	**0.265**	**0.057**	**0.215**	0.989	**-**
NA	JNJ-40418677 (GSM)	**0.282**	**0.059**	**0.208**	0.960	**-**
NA	JNJ-40418677 (GSM)	**0.273**	**0.061**	**0.222**	0.993	**-**
NA	JNJ-40418677 (GSM)	**0.280**	**0.064**	**0.228**	0.975	**-**
NA	JNJ-40418677 (GSM)	**0.260**	**0.058**	**0.222**	0.963	**-**
